# Antihyperlipidemic efficacy of aqueous extract of *Stevia rebaudiana* Bertoni in albino rats

**DOI:** 10.1186/s12944-018-0810-9

**Published:** 2018-07-27

**Authors:** Uswa Ahmad, Rabia Shabir Ahmad, Muhammad Sajid Arshad, Zarina Mushtaq, Syed Makhdoom Hussain, Aneela Hameed

**Affiliations:** 1 Department of Food Science, Nutrition & Home Economics, Government College University, Faisalabad, Pakistan; 2 Institute of Home and Food Sciences, Faculty of Science and Technology, Government College University, Faisalabad, 38000 Pakistan; 3 Department of Zoology, Government College University, Faisalabad, 38000 Pakistan; 4 Department of Food Science and Technology, Bahauudin Zakariya University, Multan, 38000 Pakistan

**Keywords:** *Stevia rebaudiana* Bertoni-Stevioside-hyperlipidemia

## Abstract

**Background:**

Stevia (*Stevia rebaudiana* Bertoni) natural, safe, non-toxic, non-caloric sugar substitute is rich source of pharmacologically important glycoside stevioside that is linked to the pathology and complications of hyperlipidemia.

**Methods:**

The present research was carried out to explore the anti-hyperlipidemic effect of aqueous extract of *Stevia rebaudiana* Bertoni leaves in albino rats. For this purpose, hyperlipidemia was induced by administration of Cholesterol (90% E, Appli Chem, Darmstadt, Germany) mixed at dose of 400 mg/kg body weight of rats in their daily routine feed. The hyperlipidemic rats were administered with aqueous stevia extract at different dose levels (200, 300, 400 and 500 ppm/kg b.w.) for 8 weeks; the control rats were fed basal diet during this period. Ethical approval for the current research was obtained from Institutional Review Board Faculty of Science & Technology Government College University, Faisalabad, Pakistan.

**Results:**

Stevia aqueous extract decreased the body weight gain by lowering the feed intake of hyperlipidemic rats. Furthermore, administration of stevia extract at different levels significantly (*P* < 0.05) lowered the TC (125.22 ± 5.91 to 110.56 ± 5.81 mg/dL), TG (102.13 ± 6.89 to 98.62 ± 7.22 mg/dL), LDL (33.02 ± 4.79 to 22.77 ± 4.36 mg/dL), VLDL (21.22 ± 5.79 to 19.33 ± 5.95 mg/dL) levels and LDL/HDL ratios (0.83 ± 1.22 to 0.54 ± 1.66 mg/dL) from H_1_ to H_4_. Conversely, it improved the HDL (39.76 ± 4.34 to l42.02 ± 4.39 mg/dL) level in hyperlipidemic rats compared with untreated rats after eight weeks study period.

**Conclusion:**

It is concluded that aqueous extract of stevia has anti-hyperlipidemic effects in albino rats, and therefore could be a promising nutraceutical therapy for the management of hyperlipidemia and its associated complications.

## Background

Hyperlipidemia is a heterogeneous disorder characterized by an elevation of total cholesterol, triglycerides, very low density lipoprotein cholesterol, low-density lipoprotein cholesterol, free fatty acids and apolipoprotein B levels, as well as reduced high-density lipoprotein cholesterol levels [1]. Among these, hypercholesterolemia and hypertriglyceridemia are closely related to ischemic heart disease. Hyperlipidemia is a common predicament in society due to change of lifestyle and food practice. Proper diet containing low fat, exercise and medication plays an important role in the prevention and treatment of increased lipid profile. Moreover, synthetic drugs are mostly used for the management of hyperlipidemia but consumption of these drugs for long period of time results in health problems such as diarrhea, liver and kidney problems due to their toxic effect. Therefore, people are more interested in using traditional medicinal plants due to their natural origin, safe and non-toxic nature [2]. *Stevia rebaudiana* Bertoni (family Asteraceae) popularly known as stevia, sweet weed, honey leaf and sweet herb of Paraguay [3]. It is natural, safe, non-toxic, non-calorie medicinal herb that has hypolipidemic ability due to presence of glycosides including stevioside, steviolbioside, rebaudiosides (A, B, C, D, E) and dulcoside A but the major sweet constituents are stevioside and rebaudioside A [4, 5]. Natural non-caloric sweetener stevioside (a major component of stevia) is 100–300 times sweeter than sucrose and have been extensively used as a non-caloric sugar substitute in many kinds of foods, medicine, beverage, cosmetics, wine making, household chemical industry and other food industries [6]. Besides hypolipidemic effect, it also posses anti-hyperglycaemic, anti-hypertensive, anti-oxidant, anti-tumor, anti-diarrheal, diuretic, gastro- and renal-protective anti-viral and immunomodulatory properties [7]. The hypolipidemic effect of stevia has been proven in both humans and rats. According to previous literature [8, 9] stevia extract has ability to reduce total cholesterol, triglycerides, low density lipoprotein and very low density lipoprotein. Moreover, it increased the level of high density lipoprotein.

As the *Stevia rebaudiana* Bertoni is safe and non-toxic natural herb and can be a better alternative of synthetic medicines used for the treatment of hyperlipidemia. Hence, the current research was carried out to investigate the hypolipidimic potential of *Stevia rebaudiana* Bertoni in albino rats.

## Methods

### Collection of material

Stevia (*Stevia rebaudiana* Bertoni*)* leaves were collected from Ayub Agricultural Research Institute (AARI), Faisalabad, Pakistan.

### Procurement of raw material

Stevia (*Stevia rebaudiana* Bertoni*)* leaves were washed to remove the dirt, dust and foreign material present on the surface. After washing, leaves of stevia were spread on trays and dried under shade at room temperature ranged from to 25–30 °C for 24–48 h. Then dried leaves were grinded into fine powder with the help of grinder **(**MJ-176-NR-3899) [10].

### Preparation of stevia aqueous extract

Stevioside was extracted from the dried ground stevia leaves by using water extraction. The dried ground leaves of stevia were mixed with hot water (65 °C) at the ratio of 1:45 (*w*/*v*) and extracted for 3 h. The crude extract containing stevioside were filtered through What man No. 1 filter paper and then evaporated to dryness by using rotary vacuum evaporator (EYELA N-1110 S 115 V) at 40–45 °C [11].

### Experimental animals

Sixty adult male albino rats of average weight 153.88 g were purchased from National Institute of Health, Islamabad, Pakistan and kept in stainless steel cages under standard conditions (temperature 25 ± 2 °C and 60 ± 5% relative humidity with 12 h light-dark cycle) in environmentally controlled animal house of college of pharmacology, Faculty of Science and Technology, Government College University Faisalabad Pakistan. The rats were acclimatized by feeding freshly prepared basal diet containing 65% starch, 10% casein, 10% corn oil, 4% salt mixture, 1% vitamins mixture and 10% cellulose [12] and distilled water for two weeks.

### Induction of hyperlipidemia

Hyperlipidemia was induced in albino rats with cholesterol (Cholesterol 90% E, Appli Chem, Darmstadt, Germany) which was mixed at dose of 400 mg/kg body weight of rats in their daily routine feed. All the experimental groups were fed on high cholesterol feed (Normal rat feed + Cholesterol) for first 15 days. H_0_ (hyperlipidemic control group) was kept on high cholesterol feed while normal control group rats (N_0_) fed on standard basal diet and distilled water throughout the experimental period (8 weeks) [13]. For the experiment stevia aqueous extract at the dose levels of 200, 300, 400 and 500 ppm/kg body weight was dissolved in the distilled water of treated hyperlipidemic rats groups and given them orally with graduated feeding bottle on daily basis.

### Animal groups and experimental design

Sixty male albino rats were divided into six groups of ten animals each and aqueous stevia extract was added in the distilled water of rats at different substitution levels given in Table 1.

**Table 1:** Diet plans for normal and hyperlipidemic rats

Normal rats	Hyperlipidemic rats				
N_0_	H_0_	H_1_	H_2_	H_3_	H_4_
(Basal diet + distilled water)	(High cholesterol diet + distilled water)	High cholesterol diet + 200 ppm SAE	High cholesterol diet+ 300 ppm SAE	High cholesterol diet + 400 ppm SAE	High cholesterol diet + 500 ppm SAE

N_0_ = Basal diet and distilled water

H_0_ = High cholesterol diet and distilled water

H_1_ = High cholesterol diet and distilled water with 200 ppm Stevia leaf extract

H_2_ = High cholesterol diet and distilled water with 300 ppm Stevia leaf extract

H_3_ = High cholesterol diet and distilled water with 400 ppm Stevia leaf extract

H_4_ = High cholesterol diet and distilled water with 500 ppm Stevia leaf extract

### Physical parameters

#### Feed and water intake

Net feed intake of individual rat was calculated on daily basis by excluding left-over and collected spilled diet during the entire period to determine the effect of individual experimental diet. Water was provided with the help of graduated drinking bottles and its consumption was also measured on daily basis.

#### Gain in body weight

Gain in body weight of individual rat in each group was estimated on weekly basis throughout the study period to find out the effect of treatments on body weight using electronic weighing balance (KERN 440-35 N).

#### Collection of serum of rats

After the 8 weeks of study period, the overnight fasted albino rats were killed using urethane anesthesia. The blood was collected by cardiac puncture and was centrifuged in the centrifuge machine (LABCENT 5000) at 3000 rpm for 15 min after allowing the blood to stand for at least 30 min at room temperature as explained by [14].

#### Serum lipid profile

Serum lipid profile including total cholesterol, triglycerides, high density lipoproteins, low density lipoproteins, very low density lipoproteins and LDL/HDL ratio were measured by using auto chemistry analyzer (Rayto RT 9200) in order to observe the variation in plasma lipid profile due to administration of aqueous stevia extract according to their respective protocols. The detail of their procedures is given below:

#### Total cholesterol level

Serum cholesterol level was determined using CHOD–PAP method following the method of [15].

#### Triglycerides level

Total triglycerides in all serum samples were determined by liquid triglycerides (GPO–PAP) method as outlined by [16].

#### High density lipoprotein level

High density lipoprotein (HDL) in serum samples was measured by HDL Cholesterol Precipitant method as mentioned by [17].

#### Low density lipoprotein and very low density lipoproteins levels

Low-density lipoproteins (LDL) and very low-density lipoproteins (VLDL) levels were calculated by using the Friedewald formula [18] as follows:

- LDL = TC–(HDL + VLDL)
- VLDL = TRIG/5

#### LDL/HDL ratio

Effect of stevia aqueous extract on LDL/HDL ratio of rats was also observed by dividing LDL and HDL.

#### Statistical analysis

The data regarding results of present research was statistically analyzed using mixed model (general linear model) by analysis of variance (ANOVA) using Minitab 17 software package. The level of significance between the mean values of samples was determined by least significant difference (LSD) [19].

## Results

### Physical parameters

It is apparent from the results that different levels of stevia aqueous extract and study period (8 weeks) significantly affected feed intake, water intake and gain in body weight of normal and hyperlipidemic albino rats.

### Feed intake

**Fig. 1:**
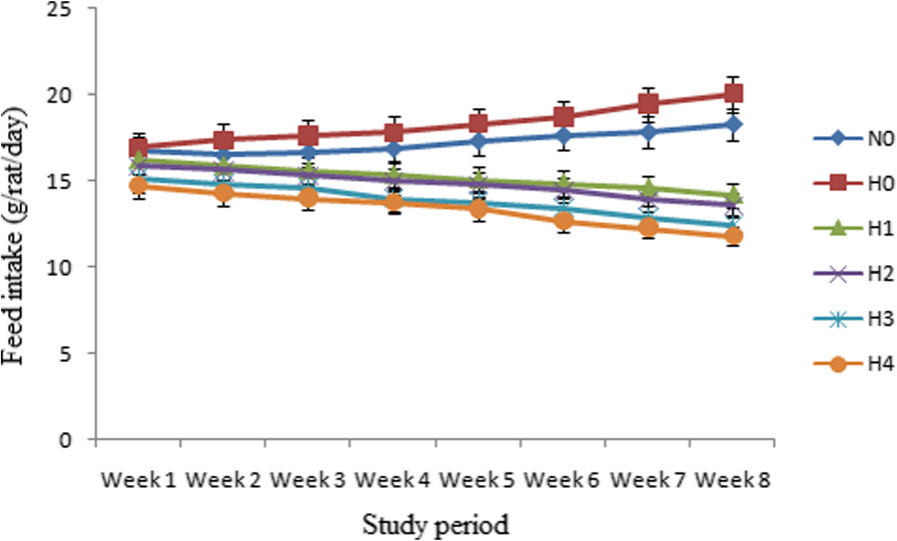
Graphical presentation of feed intake (g/rat/day) in normal and hyperlipidemic rats after 8 weeks. Results are expressed as amount of feed intake levels of hyperlipidemic rats (mean± S.E.M., *n* = 10) significantly (*P* < 0.05) different from normal and hyperlipidemic control groups

Mean values for feed intake in different groups of rats (g/rat/day) have been shown graphically in Fig. 1. The results demonstrated that administration of stevia sweetener reduced the feed intake in hyperlipidemic rats as compared to normal and hyperlipidemic groups. According to results feed intake of N_0_ and H_0_ increased from 16.72 ± 1.28 and 17.00 ± 1.30 g/rat/day at 1st week to 18.32 ± 1.76 and 20.05 ± 1.54 g/rat/day respectively at 8th week. In the case of stevia aqueous extract treated hyperlipidemic groups, the feed intake decreased as function of time and at 1st week feed intake in H_1,_ H_2_, H_3_ and H_4_ was 16.22 ± 1.14, 15.87 ± 1.43, 15.12 ± 1.04 and 14.72 ± 1.22 g/rat/day that decreased to 14.20 ± 1.02, 13.62 ± 1.23, 12.44 ± 1.00 and 11.82 ± 1.32 g/rat/day respectively at 8th week.

### Water intake

**Fig. 2:**
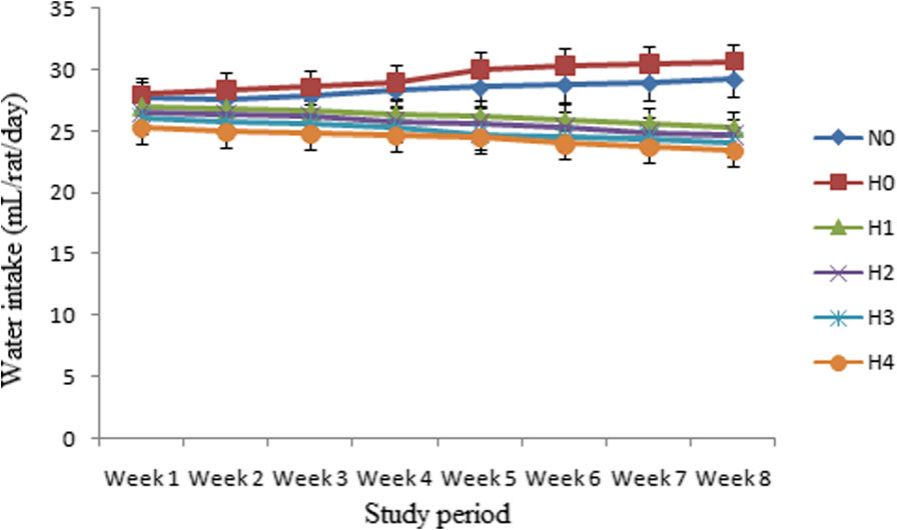
Graphical presentation of water intake (mL/rat/day) in normal and hyperlipidemic rats after 8 weeks. Results are expressed as amount of water intake levels of hyperlipidemic rats (mean± S.E.M., *n* = 10) significantly (*P* < 0.05) different from normal and hyperlipidemic control groups

Means belonging to water intake as presented in Fig. 2, showed that at 1st week it was 27.7 ± 1.01 and 27.97 ± 0.98 mL/rat/day in N_0_ and H_0_ that increased to 29.21 ± 0.34 and 30.62 ± 1.04 mL/rat/day, correspondingly at 8th week. While in H_1_, H_2_, H_3_ and H_4_ the water intake decreased from 27.00 ± 1.03, 26.50 ± 0.92, 25.80 ± 0.57 and 25.22 ± 0.87 mL/rat/day at 1st week to 25.32 ± 0.99, 24.7 ± 0.87, 24.15 ± 0.45 and 23.4 ± 0.76 mL/rat/day at 8th week respectively.

### Body weight gain

Effect of administration of stevia sweetener on the weight gain in rats has been shown in Table 2. It is apparent from the results that the highest gain in body weight was observed in hyperlipidemic group (H_0_) from 158.64 ± 4.32 g/rat at 1st week to 195.26 ± 4.50 g/rat at 8th week. While the lowest gain in body weight (150.22 ± 6.30 to 124.77 ± 7.80 g/rat) was observed in H_4_ (rats received 500 ppm/kg b.wt stevia aqueous extract) followed by H_1,_ H_2_ and H_3_ from 1st to 8 weeks.

**Table 2:** Effect of *stevia* aqueous extract on body weight (g/rat) of hyperlipidemic and normal rats

Diet groups	Week 1	Weeks 2	Weeks 3	Weeks 4	Weeks 5	Weeks 6	Weeks 7	Weeks 8
N_0_	156.28 ± 4.00Bh	160.38 ± 5.75Bg	164.14 ± 8.12Bf	168.16 ± 9.20Be	172.58 ± 9.30 Bd	176.36 ± 9.20Bc	181.38 ± 5.4Bb	186.31 ± 6.8Ba
H_0_	158.64 ± 4.32 Ah	164.77 ± 5.40Ag	169.49 ± 8.30Af	174.46 ± 8.77Ae	179.64 ± 9.11Ad	185.02 ± 7.90Ac	190.42 ± 4.3Ab	195.26 ± 4.5Aa
H_1_	153.22 ± 4.04Ca	150.42 ± 5.20Ca	147.20 ± 9.02Cb	143.54 ± 8.32Cbc	140.89 ± 9.20Cc	138.76 ± 8.78Ccd	137.89 ± 4.7Cd	134.55 ± 4.42Cd
H_2_	152.30 ± 6.30Ca	149.21 ± 4.30Ca	145.43 ± 8.44Db	142.17 ± 7.60Dbc	138.54 ± 8.62Dc	135.43 ± 9.24Dcd	132.00 ± 4.4Dd	130.22 ± 4.50Dd
H_3_	151.42 ± 5.80 Da	148.51 ± 4.76 Da	144.21 ± 5.06Db	141.65 ± 5.04Dbc	137.32 ± 7.44Dc	134.65 ± 8.99Dcd	130.76 ± 4.8Ed	126.48 ± 6.12Ed
H_4_	150.22 ± 6.30 Da	147.18 ± 3.24 Da	144.45 ± 5.02Db	140.32 ± 5.05Ebc	135.76 ± 6.65Ec	133.90 ± 7.65Ecd	129.22 ± 4.60Fd	124.77 ± 7.80Fd

Values are mean ± standard error (*n* = 10)

Mean followed by different upper case letters in the same columns represent significant difference (*P* < 0.05) treatment wise

Mean followed by different lower case letters in the same rows represent significant difference (*P* < 0.05) among study periods (8 weeks)

The results regarding body weight gain percentage (BWG %) depicted that the highest BWG % (26.95%) was observed in H_0._ On the other hand, when hyperlipidemic rats were given stevia sweetener at doses of 200, 300, 400 and 500 ppm/kg b. wt then their body weight gain BWG % decreased by − 13.58, − 15.44, − 17.89 and − 18.47% respectively after eight weeks (Table 3).

**Table 3:** Means of initial body weight, final body weight and body weight gain % in control groups rats and rats treated with different levels of stevia sweetener after 8 weeks

Experimental groups	Initial body weight (g)	Final body weight (g)	Body weight gain (%)
N_0_	152.70 ± 2.32a	186.31 ± 6.80b	22.01b
H_0_	153.80 ± 3.04a	195.26 ± 4.50a	26.95a
H_1_	155.70 ± 3.65a	134.55 ± 4.42c	−13.58c
H_2_	154.01 ± 4.02a	130.22 ± 4.50c	−15.44 cd
H_3_	154.05 ± 4.70a	126.48 ± 6.12 cd	−17.89d
H_4_	153.05 ± 4.44a	124.77 ± 7.80d	−18.47d

Results are expressed as percentage of body weight gain of hyperlipidemic and normal rats (mean ± S.E.R., *n* = 10)

N_0_ = Basal diet and distilled water

H_0_ = High cholesterol diet and distilled water

H_1_ = High cholesterol diet and distilled water with 200 ppm aqueous stevia leaf extract

H_2_ = High cholesterol diet and distilled water with 300 ppm aqueous stevia leaf extract

H_3_ = High cholesterol diet and distilled water with 400 ppm aqueous stevia leaf extract

H_4_ = High cholesterol diet and distilled water with 500 ppm aqueous stevia leaf extract

### Serum lipid profile

#### Total cholesterol level

Table 4 shows that stevia aqueous extract significantly (*P* < 0.05) affected the total cholesterol levels of hyperlipidemic rats. The results found that highest value was observed in hyperlipidemic group (H_0_) (150.55 ± 7.83 mg/dL). While total cholesterol of hyperlipidemic rats treated with stevia significantly reduced to 125.22 ± 5.91 mg/dL in H_1_, 121.63 ± 56.81 mg/dL in H_2_, 116.17 ± 5.89 mg/dL in H_3_ and 110.56 ± 5.81 mg/dL in H_4_. It is obvious from results that stevia aqueous extract decreased the cholesterol levels by 2.96, 5.74, 9.98 and 14.32% in H_1_, H_2_, H_3_ and H_4_ when rats were given stevia sweetener at doses of 200, 300, 400 and 500 mg/kg/b. wt, respectively at eight weeks study period.

**Table 4:** Mean values for serum lipid profile (mg/dL) of normal and hyperlipidemic rats after 8 weeks

Experimental groups	Total cholesterol	HDL	LDL	VLDL	LDL/HDL	Triglycerides
N_0_	129.05 ± 6.84b	41.52 ± 2.54a	35.34 ± 2.78b	22.18 ± 4.7b	0.85 ± 0.42b	103.9 ± 4.83b
H_0_	150.55 ± 7.83a	37.04 ± 3.74d	55.49 ± 3.88a	26.21 ± 6.7a	1.49 ± 0.73a	107.9 ± 5.88a
H_1_	125.22 ± 5.91c	39.76 ± 4.34c	33.02 ± 4.79c	21.22 ± 5.7c	0.83 ± 1.22c	102.13 ± 6.89c
H_2_	121.63 ± 6.81d	40.13 ± 4.74bc	30.92 ± 4.72d	20.72 ± 8.7d	0.77 ± 1.42d	101.12 ± 6.71c
H_3_	116.17 ± 5.89e	41.14 ± 4.38ab	27.82 ± 4.40e	20.56 ± 7.de	0.67 ± 1.50e	99.11 ± 6.54d
H_4_	110.56 ± 5.81f	42.02 ± 4.39a	22.77 ± 4.36f	19.33 ± 5.9e	0.54 ± 1.66f	98.62 ± 7.22d

Values with different superscript letters along the column differ significantly (*P* < 0.05)

*HDL* High density lipoprotein, *LDL* Low density lipoprotein, *VLDL* Very low density lipoprotein

N_0_ = Basal diet and distilled water

H_0_ = High cholesterol diet and distilled water

H_1_ = High cholesterol diet and distilled water with 200 ppm aqueous stevia leaf extract

H_2_ = High cholesterol diet and distilled water with 300 ppm aqueous stevia leaf extract

H_3_ = High cholesterol diet and distilled water with 400 ppm aqueous stevia leaf extract

H_4_ = High cholesterol diet and distilled water with 500 ppm aqueous stevia leaf extract

#### Triglyceride level

The mean values for triglyceride levels in normal and hyperlipidemic groups are given in Table 4. The results confirmed that highest value of triglycerides was found in H_0_ (107.90 ± 5.88 mg/dL), while lowest value was observed in H_4_ (98.62 ± 7.22 mg/dL) followed by other groups (H_1_, H_2_ and H_3_. It is confirmed from the results that triglycerides levels of hyperlipidemic rats received stevia aqueous extract at levels of 200, 300, 400 and 500 mg/kg/b. wt in H_1_, H_2_, H_3_ and H_4_ decreased by 0.93, 1.91, 3.86 and 4.33% respectively as compared to normal group (N_0_) at eight weeks study period.

#### High density lipoprotein level

The mean values for HDL as presented in Table 4 showed that level of high density lipoprotein in hyperlipidemic rats was significantly affected by different levels of stevia aqueous extracts. According to results lowest value of HDL was observed in H_0_ while that value increased in H_1_ (39.76 ± 4.34 mg/dL), H_2_ (40.13 ± 4.74 mg/dL)_,_ H_3_ (41.14 ± 4.38 mg/dL) and H_4_ (42.02 ± 4.39 mg/dL) with increasing the concentration of stevia aqueous extracts.

#### Low density lipoprotein level

The data presented in Table 4 indicated that low density lipoprotein (LDL) levels in hyperlipidemic rats were significantly (*P* < 0.05) affected by different levels of aqueous stevia extract. According to results, highest value for LDL was observed in H_0_ (55.49 ± 3.88 mg/dL), while lowest value was recorded in H_4_ (22.77 ± 4.36 mg/dL) as compared to H_1_, H_2_ and H_3_. Furthermore, the results revealed that LDL levels in H_1_, H_2_, H_3_ and H_4_ decreased by 6.56, 12.50, 21.27, 35.56% respectively as compared to normal group (N_0_) (Table 4).

#### Very low density lipoprotein level

Table 4 illustrated that very low density lipoprotein (VLDL) level in different groups of rats was considerably affected by treatments. The results confirmed that the highest value of VLDL was observed in hyperlipidemic group (H_0_). However very low density lipoprotein levels of hyperlipidemic rats administrated with stevia decreased (21.22 ± 5.79 mg/dL) in H_1_, (20.72 ± 8.79 mg/dL) in H_2_, 20.56 ± 7.75 mg/dL) in H_3_ and (19.33 ± 5.95 mg/dL) in H_4_. The results depicted that stevia aqueous extract decreased the VLDL levels in H_1_, H_2_, H_3_ and H_4_ by 16.19, 20.87, 26.39 and 31.12% respectively as compared to normal group rats (N_0_).

#### LDL/HDL ratio

The mean values for LDL/HDL ratios (Table 4) in normal and hyperlipidemic groups demonstrated that the highest value of LDL/HDL ratio was found in H_0_ (1.49 ± 0.73 mg/dL). However, lowest value of LDL/HDL ratio was observed in H_4_ (0.54 ± 1.66 mg/dL) followed by H_1_, H_2_ and H_3_.

## Discussion

The results regarding feed intake of different groups of rats found that H_0_ (Hyperlipidemic group) had higher feed intake due to high-fat diet given to them that increased their energy intake and energy storage [20]. While the hyperlipidemic rats that received stevia aqueous extract consumed less feed due to stevioside presence in it that may not stimulate the appetite of rats [21]. The results of current research work is supported by the findings of [22] who reported that stevia extract may reduced the feed intake because it is low-caloric sweetener that may not increase calorie intake and don’t stimulate appetite. Furthermore, [1] demonstrated that *Portulaca oleracea* stem may reduce the feed intake of hyperlipidemic wister albino rats.

Water intake of hyperlipidemic rats reduced after administration of stevia aqueous extract due to glycoside (stevioside) in stevia extract that decreased the water consumption of rats. The findings of present study are in collaborations with work of [21] who illustrated that hyperlipidemic rats that received stevia aqueous extract consumed less water than control group rats (normal and hyperlipidemic). Afterwards, [1, 22] found that stevia aqueous extract may reduce the water intake of hyperlipidemic albino rats.

The body weight gain of different groups of rats showed that hyperlipidemic group gained higher body weight due to high fat diet (cholesterol) used to induce hyperlipidemia in the rats that increased energy intake and energy storage [20].

When hyperlipidemic rats were given stevia sweetener at doses of 200, 300, 400 and 500 ppm/kg b. wt then their body weight gain decreased. The decrease in body weight gain was due to capability of stevioside in stevia extract that decreased the food intake of rats. Furthermore, stevioside may also reduce the body weight gain by decreasing the glucose level and promote insulin sensitivity [23]. Another reason for the decrease of body weight gain was due to stevioside ability to decrease the fat absorption and lipogenic enzymes and increase the fat excretion [24]. The results of current research are in line with [25–27] who found that there is a positive association between the decrease of body weight gain and dose of stevioside given to the rats. The body weight of rats decreased by increasing the concentration of stevioside in their diets.

The results of total cholesterol levels of different groups of rats depicted that TC level of H_0_ (hyperlipidemic group) had highest value. Conversely, addition of stevia aqueous extract at different levels lowered the TC levels in H_1_, H_2_, H_3_ and H_4_. Stevia aqueous extract contained stevioside that significantly lowered total cholesterol level due to its ability to increase the bile acid excretion by preventing reabsorption from small intestine through disruption of micelle formation of bile acid. The increase in excretion of bile acid and cholesterol activates cholesterol 7α-hydroxylase that enhances the conversion of liver cholesterol to bile acid thus resulting in cholesterol reduction [28]. The present research is in accordance with [22, 29–31] according to them mechanism for reducing cholesterol level is due to the stevioside which binds the biliary or dietary cholesterol in the colon and increases the fecal excretion of the bile acids. The increased action of 3-hydroxy-3-methylglutaryl CoA reductase (HMG-CoA) may stimulate the hepatic cholesterogenesis.

The data presented in Table 4 shows that concentration level of stevia supplementation had significant factor in lowering triglycerides in hyperlipidemic rats. In current research the increased level of triglycerides in H_0_ might be due to enhanced expression of enzymes including acetyl-coenzyme A carboxylase and fatty acid synthase, involved in TG synthesis. Moreover, malic enzyme was increased that supplies NADPH for the synthesis of long-chain fatty acids.

While in hyperlipidemic rats administrated with stevia aqueous extract, the TG levels decreased due to stevioside (major glycoside in stevia) that enhance the activity of lipase enzyme produced by liver that resulted in catabolism of lipids. Low concentration of triglycerides may also due to inhibition of dietary lipid absorption in the intestine by reducing micellar solubilization of cholesterol and by increasing excretion of TG via feces [32]. The hypolipidemic property of stevia might also be explained by interaction between stevia consumption and activation of peroxisome proliferators-activated receptors (PPARs). PPARs as a regulatory factor in lipogenesis process activate the expression of the lipoprotein lipase (LPL) and apo C-II genes as well as the hepatic uptake and etherification of free fatty acids, along with increasing mitochondrial free fatty acid oxidation [30]. The results of present research are also confirmed by the research works of [22, 26, 27, 33] who found that stevioside significantly lowered the triglyceride level as compared to untreated rats due to stevioside in stevia that reduced the activity of acetyl-coenzyme A carboxylase and fatty acid synthase.

The mean values for high density lipoprotein (HDL) levels of hyperlipidemic rats illustrated that lowest HDL level was observed in hyperlipidemic group and highest value of HDL was determined in H_4_. According to findings of present research the increase in the HDL levels of hyperlipidemic rats received stevia aqueous extracts at different levels was due to stevioside in stevia aqueous extract that improved the HDL level as compared to untreated rats. HDL (good form of lipid profile) is involved in transfer of cholesterol from tissues and arteries back to liver, thus reduced deposited cholesterol in the endothelium by retrieving cholesterol from peripheral cells and other lipoproteins to the liver for excretion in the bile and prevented LDL accumulation in the walls of the arteries [22, 32, 33]. Furthermore, [22, 26] found that stevia aqueous extract increased the HDL level in albino rats due to the elevation in the lecithin cholesterol acyl transferase (LCAT) activity which may attribute to the blood lipids regulation.

Low density lipoprotein (LDL) levels of different groups of rats demonstrated that stevia aqueous extract decreased the LDL levels in hyperlipodemic rats. The stevioside in stevia aqueous extract significantly lowered the LDL levels in hyperlipidemic rats by up regulating LDL receptor. The increase in the LDL receptor improves the uptake of low density lipoprotein cholesterol from the blood circulation [33, 34]. The findings of current research are in agreement with the studies of [1, 22] who found that stevia aqueous extract and *Portulaca oleracea* L. stem lowered the LDL level in rats because stevioside in stevia aqueous extract increase the LDL receptor and modulate cholesterol metabolism.

Very Low density lipoprotein (VLDL) levels of hyperlipidemic rats decreased after the administration of stevia aqueous extract at different dose levels due to glycoside (stevioside) in stevia extract that lowered the VLDL levels in hyperlipidemic rats. The results of present research work are in line with the work of [20, 33] who demonstrated that stevioside and solanum species (egg plant) significantly decreased the VLDL. Similary, [34] depicted that methanolic leaf extract of *Stevia rebaudiana* significantly decreased the VLDL-C levels in alloxan induced diabetic mice. Furthermore, [22, 35] illustrated that stevia aqueous extract and *Portulaca oleracea* L. stem lowered the VLDL level in rats.

The mean values for LDL/HDL ratios (Table 4) in hyperlipidemic rats demonstrated that the highest value of LDL/HDL ratio was determined in hyperlipidemic group. While, LDL/HDL ratios decreased in hyperlipidemic rats received stevioside present in stevia aqueous extracts. The results of current research are in accordance with the findings of [22, 36] who found that stevia aqueous extract and two carotenoids (lycopene and β-Carotene) supplementation lowered the LDL/HDL ratio in albino rats.

## Conclusions

The current research confirms that aqueous extract from stevia leaves may decrease the body weight gain, serum cholesterol, triglycerides, low density lipoprotein, very low density lipoprotein levels and LDL/HDL ratios. On the other hand, it improved the high density lipoprotein level of hyperlipidemic rats compared with untreated rats after eight weeks study period. It is concluded that aqueous extract of stevia with concentration 500 ppm/kg body weight of rats showed best results of all the parameters determined. It is confirmed from the results that stevia extract has hypolipidemic effects in albino rats. Nowadays obesity and hyperlipidemia are major health problems worldwide. As the diet of Pakistani population contained high amount of fat that’s why they are also facing health issues like obesity, hyperlipidemia and cardiovascular diseases. Therefore aqueous extract from Stevia leaves could be used as natural anti-hyperlipidemic drug for the treatment of hyperlipidemia and its associated complications. From the present research as it is proved that *Stevia rebaudiana* Bertoni leaves are non-toxic and safe for animals so it could be used for humans as part of their diet.

## Abbreviations

BWG %: Body weight gain percentage
HDL: High density lipoprotein
LDL: Low density lipoprotein
TC: Total cholesterol
TG: Total triglycerides
VLDL: Very low density lipoprotein
